# Is It Useful to Determine the Temperature of Children for COVID-19 Screening in the Dental Setting?

**DOI:** 10.3390/jcm11040976

**Published:** 2022-02-13

**Authors:** Eliane García-Mato, Iván Varela-Aneiros, Maite Abeleira-Pazos, Mercedes Outumuro-Rial, Pedro Diz-Dios, Jacobo Limeres-Posse, Márcio Diniz-Freitas

**Affiliations:** Special Needs Unit and OMEQUI Research Group, School of Medicine and Dentistry, Health Research Institute of Santiago de Compostela (IDIS), Santiago de Compostela University, 15706 Santiago de Compostela, Spain; eliane.garma@gmail.com (E.G.-M.); ivan.varela.aneiros@gmail.com (I.V.-A.); maite.abeleira@usc.es (M.A.-P.); mercedesoutumuro@hotmail.com (M.O.-R.); pedro.diz@usc.es (P.D.-D.)

**Keywords:** COVID-19, SARS-CoV-2, triage, dental setting, pediatric dentistry

## Abstract

Background: To date, the efficacy of temperature readings of children in the dental setting for COVID-19 screening has not been evaluated. The aim of this pilot study was to assess the usefulness of forehead temperature measurements in a dental clinic for COVID-19 screening in healthy children (without systemic disease) and in children with neurodevelopmental disorders. Methods: Using an infrared thermometer, we recorded the forehead temperature of 200 pediatric patients (100 healthy children and 100 children with neurodevelopmental disorders). We performed temperature measurements “before”, “during”, and “after” the dental procedure. Oropharyngeal swabs were taken of all participants to detect SARS-CoV-2. Results: Sex, age, administration of local anesthesia, and use of rotary instrumentation did not affect the temperature values. In the children with neurodevelopmental disorders with a value of 1 on the Frankl behavior scale, the temperatures were significantly higher than in those with values of 2, 3, and 4 (*p* = 0.032, *p* = 0.029, and *p* = 0.03, respectively). The PCR for SARS-CoV-2 was positive for two patients (one healthy and the other with a neurodevelopmental disorder), whose “before” temperatures were 36.4 °C and 36.5 °C, respectively. Conclusions: Forehead temperatures increase during dental procedures and are conditioned by the patient’s behavior. An isolated temperature reading does not identify children infected by SARS-CoV-2.

## 1. Introduction

In February 2020, the World Health Organization (WHO) officially reported the Coronavirus disease 2019 (COVID-19), and, only a month later, declared the COVID-19 pandemic [1]. It is estimated that 1–5% of all cases of COVID-19 are diagnosed in those younger than 16 years, and these individuals usually have similar clinical findings to those of adults but often with a milder presentation of the disease [2]. A relevant percentage of pediatric patients are asymptomatic [3] and could, therefore, play an important role in community-based COVID-19 transmission [4], although a number of authors do not share this opinion [5].

A recently published review that compiled information on 10,251 children from 31 countries concluded that the most prevalent manifestations of COVID-19 were fever (63.3%) and cough (33.7%) [6]. After SARS-CoV-2 was detected in saliva samples, it was suggested that the dental setting was a setting of risk for contracting COVID-19 due to the close contact with patients and the potential of exposure to contaminated saliva drops and aerosols generated during dental procedures [7]. Accordingly, it was suggested that dental patients should be scrutinized through targeted COVID-19 questions and that their body temperature be measured [8]. This proposal has been seconded by numerous experts and professional associations, and its implementation has been widespread [9].

To date, the efficacy of temperature readings of children in the dental setting for COVID-19 screening has not been evaluated. Their applicability is still less predictable in children with neurodevelopmental disorders (ND) because their thermoregulation might be abnormal due to autonomous dysregulation [10] and/or due to an abnormal response to stress conditions, which can cause changes in body temperature when they express emotions [11]. The aim of this pilot study was to assess the usefulness of forehead temperature measurements in a dental clinic for COVID-19 screening in healthy children (without systemic disease) and in children with ND.

## 2. Materials and Methods

We selected a study group of convenience consisting of 200 pediatric patients (100 healthy children and 100 with ND) who visited the Odontology Unit for Patients with Special Needs and Pediatric Dentistry of the University of Santiago de Compostela (Spain) between December 2020 and February 2021. During the participant recruitment period there were no vaccines available for children. The applied exclusion criteria were as follows: symptoms suggestive of SARS-CoV-2 infection, being within an isolation period, having direct contact with a patient with COVID-19 in the past 10 days, having received nonsteroidal anti-inflammatory drugs or other type of antipyretic in the last 24 h, and refusing to participate in the study.

The following information was collected from all participants: sex, age and presence of systemic disease (ND vs. healthy). In terms of dental treatment, we recorded whether local infiltration anesthesia was administered, whether rotary instrumentation was employed, and the degree of the child’s collaboration according to the Frankl scale, which includes the following options: 1, “definitely negative”; 2, “negative”; 3, “positive”; and 4, “definitely positive” [12].

All temperature readings were made with a non-contact infrared Berrcom^®^ thermometer (Food and Drug Administration-certified JXB-178). To record the temperature, the thermometer was placed 5 cm from the central growth point of the hair (“widow’s peak”, the V-shaped point in the hairline in the center of the forehead). After remaining in the waiting room for 5–10 min, the companion’s temperature was recorded, and an initial (“before”) reading was performed on the child. A second reading was performed on the patient halfway through the dental consultation (“during”), and the last reading was taken approximately 3 min after completing the procedure (“after”).

The patients passed through a second office in which an oropharyngeal swab was taken from them; this was sent to the laboratory for processing. We performed nucleic acid extraction in a MicrolabStarlet IVD platform using the STARMag 96 × 4 Universal Cartridge Kit (Seegene, Seoul, Korea). To detect SARS-CoV-2, we applied the Allplex™ 2019-nCoV Assay (Seegene, Seoul, Korea), a multiplex one-step real-time reverse transcription (rRT)-PCR assay targeting a conserved region in the structural protein envelope E-gene for pan-Sarbecovirus detection, RNA-dependent RNA polymerase (RdRP) and nucleocapsid (N) genes specific for SARS-CoV-2. For the rRT-PCR, we employed the CFX96™ system (Bio-Rad Laboratories, Hercules, CA, USA). We analyzed the results using Seegene Viewer-specific 2019-nCoV software (Seegene).

Before the dental intervention, we informed the patients’ guardians regarding the purpose of this study and obtained their consent. Approval by the ethics committee was not necessary, given that the measure was included in the current anti-COVID-19 protocol of the University of Santiago de Compostela.

### Statistical Analysis

We used the Shapiro–Wilk test to compare the normality of the study variables. If the distribution was normal, we applied the T-test and the ANOVA test; when it was not normal, we used the Mann–Whitney U test and the Wilcoxon test. To compare the temperature values over time, we used a mixed linear model.

## 3. Results

Fifty-nine percent of the participants were boys, and 41% were girls. The patients’ mean age was 8.5 years (range, 4–16 years). We administered local infiltration anesthesia to 38% of the patients. For 39% of the patients, we used rotary instrumentation. The degree of cooperation according to the Frankl scale was one in 11 participants (5.5%, all of them with ND), two in 38 cases (19%), three in 59 cases (29.5%) and four in the remaining 92 cases (46%).

The companions’ mean temperature was 36.411 ± 0.230 °C (range, 35.700–36.900 °C). There were no significant differences between the companions’ recorded temperatures and the children’s “before” temperatures.

The mean “before”, “during”, and “after” temperatures were 36.401 ± 0.256 °C (range, 35.700–37.000 °C), 36.892 ± 0.438 °C (range, 36.000–37.900 °C), and 36.600 ± 0.349 °C (range, 36.000–38.000 °C), respectively. The “during” temperature values increased by a mean of 0.371 °C compared with the “before” readings. After completing the procedure, the temperature decreased, but the “after” temperature values were still a mean of 0.144 °C higher than those of the “before” readings. To detect differences between the “before”, “during”, and “after” temperature values, we proposed the following model: Temperature ~ Time of measurement + (1|Patient). By applying this model, we showed that there were significant differences in the temperatures depending on when they were measured (Table 1).

The “before” temperature was significantly lower in the children with ND than in the healthy children (*p* = 0.047), but there were no statistically significant differences in the “during” and “after” measurements (Table 2).

**Figure 1:**
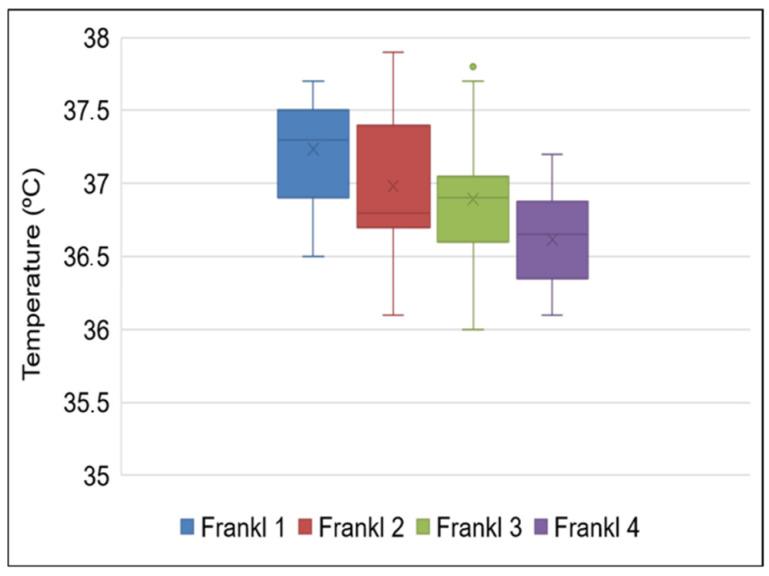
Forehead temperature values in the children with neurodevelopmental disorders in relation to the patient’s behavior, evaluated with the Frankl scale (Frankl, 1962). Frankl 1 = “definitely negative”; Frankl 2 = “negative”; Frankl 3 = “positive”; and Frankl 4 = “definitely positive”.

By applying a mixed linear model, we confirmed that the patients’ sex and age, the administration of local anesthesia, and the use of rotary instrumentation did not affect the temperature values. The patients’ behavior also did not affect the forehead temperature in the healthy children. However, in the children with neurodevelopmental disorders with a value of 1 on the Frankl behavior scale (definitely negative), the temperature readings were significantly higher than in the patients with values of 2, 3, and 4 (*p* = 0.032, *p* = 0.029 and *p* = 0.03, respectively) (Figure 1). All of these results are detailed in Table 3. 

The SARS-CoV-2 PCR was positive for two patients (one healthy and the other with an ND), whose “before” temperatures were 36.4 °C and 36.5 °C, respectively. In all of the remaining participants, the PCR was negative, including a healthy individual with a “before” temperature of 37.9 °C and another with an ND who reached 38.0 °C in the “after” measurement.

## 4. Discussion

This pilot study confirmed that forehead temperatures increase during dental procedures and that, in children with ND, the temperatures are determined by the patient’s level of behavioral control. To our knowledge, this is the first study to demonstrate that forehead temperature readings are not useful for COVID-19 triaging, either in children with ND or healthy children.

It has been confirmed that non-contact infrared thermometers are useful instruments for determining the body temperatures of children older than 1 month both in the outpatient and hospital settings [13]. However, it has been suggested that common infrared skin thermometers have a number of limitations, such as low accuracy compared with other systems like the tympanic thermometer [14], low sensitivity for detecting fever [15], and a bias related to the operator’s expertise at maintaining the thermometer at an appropriate distance from the forehead [16]. In addition, the external ambient temperature can also determine the results of the body temperature [17]. In an attempt to minimize these potential biases, we employed a calibrated thermometer, to which we incorporated a plastic rod to ensure that the recording was always performed 5 cm from the surface of the forehead, and the first recording was performed after the individual had stayed at least 5 min in the waiting room.

The reorganization of oral healthcare in the pandemic scenario (e.g., individual protective measures) has not had a profound effect on the dental anxiety of children [18]. Therefore, a higher baseline temperature in healthy children than in those with ND probably reflects a greater perception of a stressful situation such as dental treatment.

Psychological stress can trigger physiological responses, including cutaneous vasoconstriction, tachycardia, glucocorticoid release, and increased body temperature [19]. Although the mechanism of action that regulates these reactions is still unknown, a central master neuronal pathway has been described in rats that connects the circuits of corticolimbic stress to the hypothalamus and drives these autonomous and behavioral stress responses [20]. In individuals with severe intellectual disability, changes in skin temperature have been detected during the expression of emotions [11]. In the specific case of individuals with autism spectrum disorders, the atypical physiological responses to societal stimuli are determined by the severity of the individual’s autistic traits and societal skills [21]. These arguments could explain why the forehead temperatures in this study increased as behavior became poorer. The first rigorous study on the relationship between body temperature and human diseases can be traced back to end of the 19th century [22]. Before the COVID-19 pandemic, the detection of fever was one of the tools proposed for screening other severe communicable diseases such as Ebola and severe acute respiratory syndrome [23]. Temperature measurements using an infrared thermometer became a widespread recommendation for triaging COVID-19 in the dental clinic, because it is a simple, cost-effective, hygienic, and non-invasive technique [4].

The elderly was one of the most affected groups during the pivotal phase of the pandemic, and it is considered highly unlikely that a single temperature reading could detect nursing home residents infected with SARS-CoV-2 [24]. It is estimated that a single reading in the workplace would identify one case of COVID-19 for every 40 cases that would go unnoticed [25]. In the dental setting, the effectiveness of body temperature measurements and of individuals’ self-reporting of symptoms for identifying individuals infected with COVID-19 has been cast into doubt [26]. As a consequence, the measurement of absolute temperature values as a COVID-19 detection tool has been questioned [16,27,28]. It has been suggested that the difference in temperature between the forehead and other specific body locations, such as the lacrimal caruncle [29], has much more prognostic value, and the recommendation is to assess additional physiological parameters to increase the sensitivity and specificity of the noninvasive biosensors [27]. In a potential pandemic, a false negative represents false safety and a possible infection group in the future [16]. To our knowledge, this is the first study to demonstrate that forehead temperature readings do not seem to be useful for COVID-19 triaging, either in children with ND or healthy children, although the low prevalence of COVID-19 among the children included in the present series does not allow us to establish a definitive conclusion on the efficacy of this screening tool.

## Figures and Tables

**Table 1:** Differences between the forehead temperature values depending on the time at which the measurement was performed (the temperature before starting the dental procedure is taken as reference, “before”).

	Estimate	Standard Error	df	t Value	Pr(>|t|)
(Intercept)	36.401	0.036	218.169	1024.367	0.000
T “during”	0.491	0.038	198.000	12.885	0.000
T “after”	0.199	0.038	198.000	5.222	0.000

T “during”, temperature reading performed halfway through the dental procedure; and T “after”, temperature reading performed approximately 3 min after completing the procedure.

**Table 2:** Differences in the forehead temperature values between the children with neurodevelopmental disorders (ND) and the healthy children (healthy).

	Total (*n* = 200)	ND (*n* = 100)	Healthy (*n* = 100)	*p* *
**T “before”**				
Mean (SD)	36.438 (0.251)	36.401 (0.256)	36.472 (0.242)	
Median	36.400	36.300	36.500	0.047
Range	35.700–37.200	35.700–37.000	35.900–37.200	
**T “during”**				
Mean (SD)	36.866 (0.375)	36.892 (0.438)	36.842 (0.305)	
Median	36.800	36.900	36.800	0.559
Range	36.000–37.900	36.000–37.900	36.200–37.900	
**T “after”**				
Mean (SD)	36.608 (0.309)	36.600 (0.349)	36.616 (0.268)	
Median	36.600	36.600	36.600	0.236
Range	35.900–38.000	36.000–38.000	35.900–37.500	

T “before”, temperature reading performed before starting the dental procedure; T “during”, temperature reading performed halfway through the dental procedure; and T “after”, temperature reading performed approximately 3 min after completing the procedure. * Wilcoxon rank-sum test.

**Table 3:** Variables that determine the forehead temperature values applying a mixed linear model in children with neurodevelopmental disorders (ND) and in healthy children (healthy).

		Estimate	Std. Error	df	t Value	Pr(>|t|)
(Intercept)	ND Healthy	36.671 36.077	0.164 0.318	94.334 101.339	223.040 113.479	0.000 0.000
T “during”	ND Healthy	0.491 0.371	0.038 0.023	198.000 216.000	12.885 16.441	0.000 * 0.000 *
T “after”	ND Healthy	0.199 0.144	0.038 0.023	198.000 216.000	5.222 6.389	0.000 * 0.000 *
Sex	ND Healthy	−0.108 0.074	0.057 0.049	91.000 101.000	−1.902 1.530	0.060 0.129
Age	ND Healthy	0.003 0.000	0.008 0.009	91.000 101.000	0.419 −0.052	0.676 0.959
Local anesthesia	ND Healthy	−0.072 0.020	0.069 0.052	91.000 101.000	−1.044 0.382	0.299 0.703
Rotary Instrumentation	ND Healthy	0.106 −0.042	0.068 0.049	91.000 101.000	1.557 −0.853	0.123 0.396
Frankl Scale (value of 2)	ND Healthy ^‡^	−0.211 -	0.097 -	91.000 -	−2.181 -	0.032 ^†^ -
Frankl Scale (value of 3)	ND Healthy	−0.256 0.023	0.115 0.110	91.000 101.000	−2.221 0.211	0.029 ^†^ 0.833 ^#^
Frankl Scale (value of 4)	ND Healthy	−0.369 0.003	0.120 0.105	91.000 101.000	−3.076 0.024	0.003 ^†^ 0.981 ^#^

T “during”: temperature reading performed halfway through the dental procedure; T “after”: temperature reading performed approximately 3 min after completing the procedure; * The temperature before starting the dental procedure is taken as reference (“before”); ^†^ The value of 1 on the Frankl scale is taken as reference; **^‡^** Not applicable because there are no healthy childrenwith a value of 1 on the Frankl scale; ^#^ The value of 2 on the Frankl scale is taken as reference.

## Data Availability

The data that support the findings of this study are available on request from the corresponding authors.
